# Effect of Probiotic Supplementation on Reproductive Outcomes of Women With Polycystic Ovary Syndrome Who Are Candidates for Intrauterine Insemination: A Randomized, Double‐Blind, Placebo‐Controlled Trial

**DOI:** 10.1002/edm2.70185

**Published:** 2026-03-03

**Authors:** Tahereh Behroozilak, Mahsa Farshadfar, Samira Jahangard

**Affiliations:** ^1^ Reproductive Health Research Center, Clinical Research Institute Urmia University of Medical Sciences Urmia Iran; ^2^ Department of Obstetrics and Gynecology, School of Medicine Urmia University of Medical Sciences Urmia Iran; ^3^ Department of Obstetrics and Gynecology, Kosar Hospital Urmia University of Medical Sciences Urmia Iran

**Keywords:** assisted reproductive techniques, infertility, intrauterine insemination, polycystic ovary syndrome, probiotics

## Abstract

**Introduction:**

Evidence showed that microbial dysbiosis may lead to poor fertility outcomes. Today, therapy with probiotics, especially when using assisted reproductive technologies, is considered a potential method to improve outcomes. This study aimed to evaluate the effectiveness of probiotic consumption on reproductive outcomes in women with polycystic ovary syndrome (PCOS) who are candidates for Intrauterine Insemination (IUI).

**Methods:**

In this randomized, double‐blind clinical trial, 100 women aged 19 to 37 years with PCOS who were candidates for IUI were studied in two groups (1:1): intervention and placebo. After ovulation induction and IUI, the study outcomes including chemical and clinical pregnancy rates, number of dominant follicles, and endometrial thickness were examined and compared in the two groups.

**Results:**

No difference was observed between the intervention and control groups in terms of basic demographic findings and paraclinical evaluation (*p* > 0.05). The chemical pregnancy rate in the intervention group was higher than in the placebo group, but no statistically significant difference was observed (16% vs. 12%; *p* = 0.564). The clinical pregnancy rate in the intervention group was higher than in the placebo group (14% vs. 4%; *p* = 0.081). The mean endometrial thickness in intervention groups was significantly higher than placebo group (*p* = 0.028), while the mean dominant follicle between the two groups was almost the same (p > 0.05). The regression model showed that only the probiotic supplementation had a significant positive effect on endometrial thickness in the intervention than the placebo groups (*β* = 0.618, 95% CI: 0.167–1.069, *p* = 0.008).

**Conclusion:**

Probiotic supplementation for 8 weeks in women with PCOS who were candidates for IUI was associated with higher endometrial thickness, indicating a potential role in improving endometrial receptivity.

## Graphical abstract

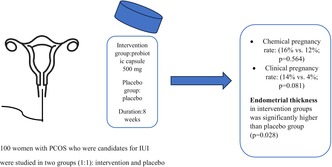

## 1 Introduction

Polycystic ovary syndrome (PCOS) is a common endocrinopathy in reproductive‐aged women [1]. Hormonal and metabolic disturbances in women with PCOS put them at increased risk of multiple reproductive, oncological, cardiometabolic, and psychological complications [2]. Since folliculogenesis and ovarian steroidogenesis are more likely to be altered in women with PCOS [3], infertility is one of the common complications among women with PCOS. Three out of every 10 infertile women suffer from PCOS [4]. Also, it accounts for 80% of anovulatory infertility cases [5].

There are multiple management strategies for infertility in women with PCOS [6]. When lifestyle modification and pharmacological ovulation induction fail in women with PCOS, assisted reproductive techniques (ART) are advised [7]. Intrauterine insemination (IUI) is one of the ART techniques that is suitable for women with PCOS who ovulate after ovulation induction or who have male or immunological factors, or cervical factors [6]. However, some evidence cannot support the beneficial effect of adding IUI ovulation induction protocols on fertility outcomes among women with PCOS [8]. IUI might be an assist for infertile women with PCOS before IVF/ICSI and might accelerate pregnancy for target women without invasive manipulation [9].

The metabolic and hormonal status in women with PCOS not only influences the chance of pregnancy but also compromises the success rates of ART [10, 11, 12]. Moreover, dysbiosis of gut microbiota as a hidden player in PCOS can also affect the fertility of these women [13]. So, emerging evidence indicates that the regulation of gut microflora has emerged as a novel management approach for PCOS [14, 15]. Numerous randomized controlled trials have investigated the effect of probiotics on different parameters of PCOS management [16, 17]. Probiotics have also the envisaged role in the treatment of infertility [18]. Previous trials have suggested that the administration of probiotics may improve the chemical and clinical pregnancy rates [19]. Moreover, probiotics have been linked to favourable changes in metabolic parameters, as well as reductions in inflammatory and oxidative stress [17, 20].

Most available studies focus on the effect of probiotics on metabolic and hormonal parameters rather than fertility outcomes in women with PCOS [16]. Despite these promising findings, evidence on the effect of probiotic supplementation on clinical outcomes following IUI in women with PCOS remains limited. Therefore, this study aimed to evaluate the effectiveness of probiotic consumption on reproductive outcomes in women with PCOS who are candidates for IUI.

## 2 Materials and Methods

This study was designed as a randomized, double‐blind, placebo‐controlled clinical study. The study protocol was approved by the research ethics committees of Urmia University of Medical Sciences (IR.UMSU.REC.1402.072). Clinical Trial number for the study: IRCTID: IRCT20160308026962N6.

### 2.1 Study Participants

In this study, 100 infertile women with PCOS who were candidates for IUI and were referred to the infertility clinic of Kowsar Hospital, Urmia, Iran were included.

Based on the study by Azizi‐Kutenaee et al. [19], which reported a clinical pregnancy rate of 0% in the placebo group and 10% in the probiotic group, and using G Power software, considering an alpha error of 5%, a power of 80%, and an Effect Size = 0.26, the sample size was calculated to be 119 cases.

Inclusion criteria include: women were at reproductive age (age 19 to 37), had a confirmed diagnosis of PCOS based on the 2003 Rotterdam criteria [21], and had infertility for at least 1 year despite unprotected intercourse.

### 2.2 Exclusion Criteria Include

Women with chronic diseases (including diabetes, lung, pancreas, kidney, liver, cardiovascular diseases, thyroid disorders, hyperprolactinemia, Cushing's syndrome, autoimmune disease), allergies to probiotics, use of antibiotics or multivitamin‐mineral supplements in the last 3 months, and patients treated with specific diets and physical activity were excluded from the study.

**FIGURE 1:**
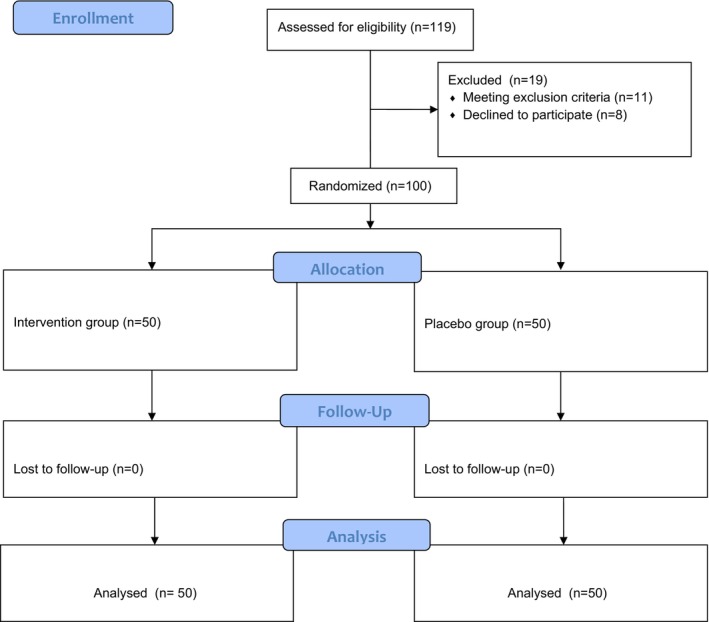
CONSORT: Flow of participants assigned to the control and intervention groups.

Figure 1 presents the flowchart illustrating the recruitment, randomization and allocation process in the study.

### 2.3 Randomization

The women were randomly assigned to one of the intervention (probiotic) or control (placebo) groups using computer‐generated random numbers and 6‐digit blocks. First, a set of 6‐digit random numbers was generated by statistical software. Then, a random number was assigned to each participant. Based on the last digit of each number, group assignment was done as follows: If the last digit was even, the participant was placed in the intervention group. If the last digit was odd, the participant was placed in the placebo group.

### 2.4 Blinding

The probiotic and placebo capsules were similar in size, tone, odour and packaging and were numbered by a pharmacist. Participants, laboratory staff and the person assessing the outcomes were blinded to the study groups.

### 2.5 Formulations and Procedures

After assessing the inclusion criteria, the patients were randomly divided into two groups of 50 (1:1) using a sealed envelope. The patients were matched in terms of age and body mass index (BMI). At enrolment, all patients underwent clinical and paraclinical evaluation before starting the IUI cycle. In patients with indications, folic acid was also prescribed daily in an amount of 400 micrograms to prepare for pregnancy. The intervention duration was 2 months.

#### 2.5.1 Intervention Group

Each probiotic capsule (500 mg; brand name LactoFem, Zist‐Takhmir) contains 7 beneficial bacterial strains including *Lactobacillus acidophilus* (ATCC: 4357) 3 × 1010 colony‐forming units (CFU), Lacticaseibacillus casei (ATCC: 393) 3 × 109 CFU, *Lactobacillus bulgaricus* (ATCC: 11842) 5 × 108 CFU, Lacticaseibacillus rhamnosus (ATCC: 7469) 7 × 109 CFU, *Bifidobacterium longum* (ATCC: 15697) 1 × 109 CFU, *Bifidobacterium breve* (ATCC: 15700) 2 × 1010 CFU and *Streptococcus thermophilus* (ATCC: 19258) 3 × 108 CFU (74).

Initially, one probiotic capsule was administered orally daily for 1 month. From the second month, ovulation‐stimulating drugs were prescribed from the third day of the menstrual cycle along with the oral probiotic capsule of the previous month.

#### 2.5.2 Placebo Group

In this group, like the intervention group, a placebo probiotic was administered, and in the second month, ovulation‐stimulating drugs were administered alone from the third day of the menstrual cycle. Each placebo capsule contained 250 mg of starch and 250 mg of maltodextrin, which was produced by the same probiotic manufacturer (Zist‐Takhmir Company) and was completely similar in size, colour and odour to the probiotic‐containing capsule.

Patients receiving either a probiotic or a placebo were instructed to store the medication at 2°C–8°C and take one capsule orally daily after meals. To increase patient compliance, they were asked to return the capsule shells after use. Patients were also asked to maintain their diet and physical activity while receiving the drug.

Patients were evaluated by vaginal ultrasound during the 12th to 16th day of the menstrual cycle for response to treatment, ovarian follicle size growth, and endometrial thickness. If at least one follicle larger than 18 mm was seen, 5000 IU of human chorionic gonadotropin (hCG) was injected intramuscularly to stimulate ovulation, and patients underwent IUI.

### 2.6 Outcomes

Primary outcomes (1) Chemical pregnancy rate: Chemical confirmation of pregnancy was defined as serum beta HCG greater than 50 IU/L, 14 days after IUI. (2) Clinical pregnancy rate: Clinical pregnancy was defined as the observation of fetal heart activity by transvaginal ultrasound 2 weeks after beta HCG became positive (i.e., 6 weeks of gestation).

Secondary outcomes (1) Number of dominant follicles: follicles larger than 18 mm. (2) Endometrial thickness (ET): assessment using transvaginal ultrasound.

### 2.7 Assessments

Fasting blood samples (10 mL) were collected at baseline Kosar hospital reference laboratory. Serum concentration of follicle stimulating hormone (FSH), luteinizing hormone (LH), thyroid stimulating hormone (TSH) and Prolactin (PRL) were measured using the enzyme‐linked immunosorbent assay (ELISA) method. Commercial ELISA kits, including Pishtaz Teb (Iran), Monobind (USA) and Diaplus (USA), were employed according to the manufacturers' instructions. All assays were performed in duplicate, and the intra‐ and inter‐assay coefficients of variation were within the acceptable range.

Ultrasonographic examinations were carried out by a gynaecologist with a Philips Affiniti 50 GI system, providing a spatial resolution accuracy of approximately ±1 mm.

Anthropometric measurements were obtained using a digital stadiometer and weighing scale (Rahbanan Sazandegi, Model C011000), with a precision of ±0.1 cm for height and ±0.1 kg for weight.

### 2.8 Statistics

The statistical analysis was performed using SPSS version 21. The level of significance was considered *p* < 0.05. The normality of the measurement distributions was assessed using the Kolmogorov–Smirnov test. Categorical variables are reported as frequencies (%) and compared between groups by the Chi‐square test or Fisher's exact test. The independent *t*‐test was used to compare quantitative characteristics between the intervention and placebo groups. In cases with non‐normal distribution, the non‐parametric tests were applied. Linear regression models were also applied to assess the association of probiotic supplementation and reproductive outcomes.

## 3 Results

All participants from both groups remaining in the study were included in the analyses. The baseline characteristics of the groups revealed no differences between placebo and probiotic groups (*p* > 0.05) (Table 1).

**TABLE 1:** Baseline characteristic of the groups (before intervention).

Variable	Intervention group (*n* = 50)	Placebo group (*n* = 50)	*p*
Age (year)	28.4 ± 4.4	29.1 ± 5.5	0.355
Duration of marriage (years)	5.8 ± 2.2	6.1 ± 2.5	0.383
Duration of infertility (years)	2.8 ± 1.1	2.7 ± 1.1	0.750
BMI (kg/m^2^)	26.1 ± 1.9	26.7 ± 2.2	0.197
TSH (mIU/L)	2.9 ± 1.2	2.9 ± 1.1	0.715
FSH (mIU/mL)	6.27 ± 1.48	6.01 ± 1.68	0.413
LH (IU/mL)	7.7 ± 3.9	8 ± 3.5	0.605
PRL (ng/mL)	100.6 ± 11.11	102.45 ± 11.69	0.400

Abbreviations: BMI, Body Mass Index; FSH, follicle stimulating hormone; LH, luteinizing hormone; PRL, Prolactin; TSH, thyroid stimulating hormone.

### 3.1 Primary Outcomes

The results showed that according to the results of the Chi‐square test, the frequency of chemical pregnancy and clinical pregnancy between the two intervention and placebo groups was almost the same and was not statistically significant (*p* > 0.05) (Table 2).

**TABLE 2:** Comparison primary outcomes between groups.

Variable		Groups		*p*
		Intervention *N* (%)	Placebo *N* (%)	
Chemical pregnancy	Positive	8 (%16)	6 (%12)	0.564
	Negative	42 (%84)	44 (%88)	
Clinical pregnancy	Positive	7 (%14)	2 (%4)	0.081

### 3.2 Secondary Outcomes

The mean endometrial thickness between the two intervention and placebo groups was not the same and was statistically significant (*p* = 0.028). Also, according to the results of the Mann–Whitney test, the mean dominant follicle between the two intervention and placebo groups was almost the same and was not statistically significant (Table 3).

**TABLE 3:** Comparison of secondary outcomes between groups.

Variable	Groups		*p*
	Intervention mean ± SD	Placebo mean ± SD	
Dominant follicle	2.1 ± 0.8	2.2 ± 0.8	0.299
Endometrial thickness (mm)	9 ± 0.7	9.6 ± 1.3	0.028

The results of the regression model showed that the probiotic supplementation only had a significant protective effect on endometrial thickness in the intervention than the placebo groups (β = 0.618, 95% CI: 0.167–1.069, *p* = 0.008) and the other variables of chemical pregnancy, clinical pregnancy and dominant follicle were not significant (Table 4).

**TABLE 4:** Linear regression model assessing the association of probiotic supplementation and reproductive outcomes.

Outcomes	β (Treatment vs Placebo)	95% confidence interval for B		*p*
		Lower	Upper	
Chemical Pregnancy	−0.031	−0.176	0.114	0.674
Clinical Pregnancy	−0.089	−0.208	0.030	0.139
Dominant follicle	0.140	−0.0183	0.464	0.392
Endometrial thickness (mm)	0.618	0.167	1.069	0.008

*Note:* All models were adjusted for relevant covariates (age, BMI, marriage duration, infertility duration, follicle stimulating hormone, luteinizing hormone, thyroid stimulating hormone, Prolactin). Only the adjusted effect of treatment is shown.

## 4 Discussion

In this study, we investigated the effect of probiotic supplementation on reproductive outcomes, including chemical pregnancy, clinical pregnancy, number of dominant follicles and endometrial thickness of women with PCOS who were candidates for IUI. Our findings demonstrated that in the group with probiotic consumption, ET was significantly higher than in the control group, whereas its effects on chemical pregnancy, clinical pregnancy and the number of dominant follicles were not statistically significant.

Recently, the role of probiotics in the management of PCOS as well as pregnancy outcome has gained more attention [22]. Women with PCOS may experience changes in the microbiome [23]. Therefore, emerging evidence suggests that modulation of the gut microbiome may be a potential therapeutic target for managing PCOS [24]. Moreover, evidence showed that female microbiota dysbiosis (vaginal, endometrial) can influence their fertility outcomes [25]. Microbial dysbiosis may lead to poor fertility outcomes, and the expert opinion recommended probiotics as a safe and non‐invasive approach in improving outcomes of ART [26]. There are rationales for supporting the role of probiotics therapy on improving reproductive health and pregnancy outcome [27]. Understanding the role of probiotics in this pathway is therefore important, as it may enhance reproductive outcomes in women undergoing IUI, particularly those with PCOS.

Although the clinical pregnancy rate was higher in the probiotic group, the difference did not reach statistical significance; this value indicates a trend towards improvement. One possible explanation is that the sample size may have been insufficient to detect a significant effect. In line with our study, a single‐blind clinical trial that evaluated the effect of probiotic supplementation on pregnancy rates in women undergoing IUI showed that the pregnancy rate in the probiotic group did not significantly differ from that in the placebo group [28]. Additionally, a study by Thanaboonyawat et al. evaluated the effect of vaginal probiotic supplementation with Lactobacillus before frozen embryo transfer on pregnancy outcomes, but it did not improve the biochemical and clinical pregnancy rates [29]. In contrast to the current study, the effect of Lactofem supplementation in women with PCOS was also investigated in the study by Azizi‐Kutenaee et al. during an 8 week intervention, and the results showed that the rates of chemical pregnancy and clinical pregnancy were significantly higher in women receiving probiotic supplementation than in the placebo group [19]. A systematic review and meta‐analysis also showed that vaginal probiotics before embryo transfer led to a non‐significant increase in clinical pregnancy rates [30]. Multiple factors, including dose, duration and type of probiotics, as well as participants' characteristics, have been shown to affect fertility outcomes in previous studies. Moreover, limited studies examined the effect of probiotics on the reproductive outcomes of women candidates for IUI. It is proposed that the IUI success rate, per se, depends on various factors like duration of infertility, sperm concentration, couple age and endometrium thickness [31].

The significant differences in endometrial thickness observed between the intervention group and the placebo group suggest that probiotics may enhance endometrial receptivity. This could be explained by the immunomodulatory [32, 33], anti‐inflammatory [34, 35] and antioxidant [36] properties of probiotics, which may be linked to improved uterine blood flow and endometrial development. A receptive endometrium is one of the critical factors for successful implantation [37], and therefore, this finding highlights a potential benefit of probiotic supplementation in IUI outcomes. Emerging evidence supports a gut–reproductive axis whereby gut microbiota modulates reproductive health via immune, endocrine, and metabolic pathways. Dysbiosis is associated with systemic inflammation, altered oestrogen metabolism and immune dysregulation—factors known to impair endometrial function and receptivity [38, 39]. A recent review highlighted the interaction between microbiota and immunity as new therapeutic opportunities [40]. Therefore, probiotic bacteria via immunomodulatory properties can improve immune functions as well as health [33]. Today, researchers emphasized that the endometrium is a biologically active microbial niche that plays a role in reproductive health [41]. Recently published Expert Opinion also supported the significant therapothic role of Probiotics in infertility via improving overall bacterial profile in women [26].

The strengths of the current study include the nature of the clinical trial, follow‐up, and collection of capsule shells to enhance patient compliance, as well as the implementation of a single protocol for IUI within a single clinical centre, which minimized the risk of bias. The probiotic used in the current intervention contained seven beneficial bacterial strains, with Lactobacillus being the predominant strain. The current study also has limitations. The main limitations of the current study include the limited sample size, the lack of measurement of serum LH, FSH, and prolactin levels after the intervention, and the lack of examination of metabolic syndrome parameters and lipid profiles in women with PCOS who were candidates for IUI. Given the near‐significant result observed for clinical pregnancy, further randomized controlled trials with larger sample sizes and longer follow‐up are necessary to confirm whether these improvements in endometrial thickness can ultimately translate into higher pregnancy success rates. Morover, profiling gut and potentially endometrial microbiota using metagenomics and metabolomics would allow correlations between microbial shifts and key reproductive outcomes including implantation success, pregnancy maintenance, and live birth. This approach would strengthen causal inference regarding probiotic effects and elucidate strain‐specific or metabolite‐mediated mechanisms. Therefore, we propose that future multicentre RCT studies adopt integrative microbiome analyses alongside clinical endpoints to advance the field.

## 5 Conclusion

This study demonstrated that in a group supplemented with probiotics, there was a significantly higher endometrial thickness, indicating a potential role in improving endometrial receptivity. However, no significant effects were observed on chemical pregnancy rate, clinical pregnancy rate or the number of dominant follicles.

## Author Contributions

Conceptualization: Tahereh Behroozilak and Mahsa Farshadfar, Data curation: Tahereh Behroozilak and Mahsa Farshadfar, Formal analysis: Tahereh Behroozilak, Investigation: Tahereh Behroozilak and Mahsa Farshadfar. Methodology: Tahereh Behroozilak and Mahsa Farshadfar, Supervision: Tahereh Behroozilak, Writing – original draft: Tahereh Behroozilak, Samira Jahangard and Mahsa Farshadfar, Writing – review and editing: Tahereh Behroozilak, Samira Jahangard, and Mahsa Farshadfar.

## Conflicts of Interest

The authors declare no conflicts of interest.

## Data Availability

The data that support the findings of this study are available on request from the corresponding author. The data are not publicly available due to privacy or ethical restrictions.

## References

[1] R. Deswal , V. Narwal , A. Dang , and C. S. Pundir , “The Prevalence of Polycystic Ovary Syndrome: A Brief Systematic Review,” Journal of Human Reproductive Sciences 13, no. 4 (2020): 261–271, 10.4103/jhrs.JHRS_95_18. 33627974 PMC7879843

[2] S. Palomba , S. Santagni , A. Falbo , and G. B. La Sala , “Complications and Challenges Associated With Polycystic Ovary Syndrome: Current Perspectives,” International Journal of Women's Health 7 (2015): 745–763, 10.2147/ijwh.S70314. PMC4527566 26261426

[3] C. R. V. Leal , K. Zanolla , P. M. Spritzer , and F. M. Reis , “Assisted Reproductive Technology in the Presence of Polycystic Ovary Syndrome: Current Evidence and Knowledge Gaps,” Endocrine Practice 30, no. 1 (2024): 64–69, 10.1016/j.eprac.2023.09.004. 37708997

[4] Z. Alam , S. Alseari , M. Alameemi , et al., “Prevalence of Polycystic Ovary Syndrome Among Infertile Women in the Gulf Cooperation Council (GCC) Countries: A Systematic Review and Meta‐Analysis,” Heliyon 10, no. 24 (2024): e40603, 10.1016/j.heliyon.2024.e40603. 39759288 PMC11700273

[5] A. S. Melo , R. A. Ferriani , and P. A. Navarro , “Treatment of Infertility in Women With Polycystic Ovary Syndrome: Approach to Clinical Practice,” Clinics (São Paulo, Brazil) 70, no. 11 (2015): 765–769, 10.6061/clinics/2015(11)09. 26602525 PMC4642490

[6] M. Li , X. Ruan , and A. O. Mueck , “Management Strategy of Infertility in Polycystic Ovary Syndrome,” Global Health Journal 6, no. 2 (2022): 70–74, 10.1016/j.glohj.2022.03.002.

[7] A. Cunha and A. M. Póvoa , “Infertility Management in Women With Polycystic Ovary Syndrome: A Review,” Porto Biomedical Journal 6, no. 1 (2021): e116, 10.1097/j.pbj.0000000000000116. 33532657 PMC7846416

[8] J. Aly , M. B. Evans , S. Jahandideh , A. Decherney , K. Devine , and M. Hill , “The Utility of Intra‐Uterine Insemination in the Treatment of Polycystic Ovarian Syndrome,” Fertility and Sterility 113, no. 4 (2020): e44–e45.

[9] Y. Gao , S. Jiang , L. Chen , et al., “The Pregnancy Outcomes of Infertile Women With Polycystic Ovary Syndrome Undergoing Intrauterine Insemination With Different Attempts of Previous Ovulation Induction,” Front Endocrinol (Lausanne) 13 (2022): 922605, 10.3389/fendo.2022.922605. 36093093 PMC9450480

[10] M. N. Gunning , J. P. Christ , B. B. van Rijn , et al., “Predicting Pregnancy Chances Leading to Term Live Birth in Oligo/Anovulatory Women Diagnosed With PCOS,” Reproductive Biomedicine Online 46, no. 1 (2023): 156–163, 10.1016/j.rbmo.2022.09.024. 36411204

[11] L. Wang , X. Yu , D. Xiong , et al., “Hormonal and Metabolic Influences on Outcomes in PCOS Undergoing Assisted Reproduction: The Role of BMI in Fresh Embryo Transfers,” BMC Pregnancy and Childbirth 25, no. 1 (2025): 368, 10.1186/s12884-025-07480-9. 40155948 PMC11951658

[12] H. J. Guan , L. Q. Pan , H. Song , H. Y. Tang , and L. S. Tang , “Predictors of Pregnancy After Intrauterine Insemination in Women With Polycystic Ovary Syndrome,” Journal of International Medical Research 49, no. 5 (2021): 3000605211018600, 10.1177/03000605211018600. 34038202 PMC8161844

[13] H. Senthilkumar and M. Arumugam , “Gut Microbiota: A Hidden Player in Polycystic Ovary Syndrome,” Journal of Translational Medicine 23, no. 1 (2025): 443, 10.1186/s12967-025-06315-7. 40234859 PMC11998441

[14] A. Arab , M. Hossein‐Boroujerdi , A. Moini , M. Sepidarkish , N. Shirzad , and E. Karimi , “Effects of Probiotic Supplementation on Hormonal and Clinical Outcomes of Women Diagnosed With Polycystic Ovary Syndrome: A Double‐Blind, Randomized, Placebo‐Controlled Clinical Trial,” Journal of Functional Foods 96 (2022): 105203, 10.1016/j.jff.2022.105203.

[15] H. Ramzan , D. A. Bukhari , Z. Bibi , A. Nawaz , and A. Rehman , “Probiotic Supplement for the Treatment of Polycystic Ovarian Syndrome,” Pharmacology and Therapeutics 266 (2025): 108785, 10.1016/j.pharmthera.2024.108785. 39719172

[16] D. Martinez Guevara , S. Vidal Cañas , I. Palacios , et al., “Effectiveness of Probiotics, Prebiotics, and Synbiotics in Managing Insulin Resistance and Hormonal Imbalance in Women With Polycystic Ovary Syndrome (PCOS): A Systematic Review of Randomized Clinical Trials,” Nutrients 16, no. 22 (2024): 3916. 39599701 10.3390/nu16223916 PMC11597640

[17] M. Cozzolino , A. Vitagliano , L. Pellegrini , et al., “Therapy With Probiotics and Synbiotics for Polycystic Ovarian Syndrome: A Systematic Review and Meta‐Analysis,” European Journal of Nutrition 59, no. 7 (2020): 2841–2856, 10.1007/s00394-020-02233-0. 32372265

[18] N. Younis and A. Mahasneh , “Probiotics and the Envisaged Role in Treating Human Infertility,” Middle East Fertility Society Journal 25, no. 1 (2020): 33, 10.1186/s43043-020-00039-y. PMC7542571 33046958

[19] M. Azizi‐Kutenaee , S. Heidari , S.‐A. Taghavi , and F. Bazarganipour , “Probiotic Effects on Sexual Function in Women With Polycystic Ovary Syndrome: A Double Blinded Randomized Controlled Trial,” BMC Women's Health 22, no. 1 (2022): 373. 36096842 10.1186/s12905-022-01955-z PMC9465857

[20] I. Szydłowska , J. Nawrocka‐Rutkowska , A. Gorzko , H. Pawłowski , A. Starczewski , and M. Szczuko , “Changes in Hormonal Profile and Body Mass Index in Women With Polycystic Ovary Syndrome After Probiotic Intake: A 12‐Week Placebo‐Controlled and Randomized Clinical Study,” Nutrients 17, no. 3 (2025): nu17030405, 10.3390/nu17030405. PMC11820849 39940263

[21] Group REASPCW , “Revised 2003 Consensus on Diagnostic Criteria and Long‐Term Health Risks Related to Polycystic Ovary Syndrome (PCOS),” Human Reproduction 19, no. 1 (2004): 41–47. 14688154 10.1093/humrep/deh098

[22] L.‐Y. Wu , T.‐H. Yang , Y.‐C. Ou , and H. Lin , “The Role of Probiotics in Women's Health: An Update Narrative Review,” Taiwanese Journal of Obstetrics & Gynecology 63, no. 1 (2024): 29–36, 10.1016/j.tjog.2023.09.018. 38216265

[23] A. Sola‐Leyva , I. Pérez‐Prieto , N. M. Molina , et al., “Microbial Composition Across Body Sites in Polycystic Ovary Syndrome: A Systematic Review and Meta‐Analysis,” Reproductive Biomedicine Online 47, no. 1 (2023): 129–150, 10.1016/j.rbmo.2023.03.016. 37208218

[24] M. G. Rizk and V. G. Thackray , “Intersection of Polycystic Ovary Syndrome and the Gut Microbiome,” Journal of the Endocrine Society 5, no. 2 (2020): bvaa177, 10.1210/jendso/bvaa177. 33381671 PMC7757431

[25] M. A. Venneri , E. Franceschini , F. Sciarra , E. Rosato , G. D'Ettorre , and A. Lenzi , “Human Genital Tracts Microbiota: Dysbiosis Crucial for Infertility,” Journal of Endocrinological Investigation 45, no. 6 (2022): 1151–1160, 10.1007/s40618-022-01752-3. 35113404 PMC9098539

[26] A. Patki , S. Kar , N. Patel , et al., “Expert Opinion: Place in Therapy of Probiotics in Infertility and Recurrent Implantation Failure,” Cureus 17, no. 3 (2025): e81067. 40271314 10.7759/cureus.81067 PMC12016387

[27] J. N. Reid , J. E. Bisanz , M. Monachese , J. P. Burton , and G. Reid , “The Rationale for Probiotics Improving Reproductive Health and Pregnancy Outcome,” American Journal of Reproductive Immunology 69, no. 6 (2013): 558–566. 23414386 10.1111/aji.12086

[28] Z. Rezaei , K. Adabi , E. Feizabad , and M. Aliakbar , “The Effect of Probiotic Supplementation in Intrauterine Sperm Insemination Pregnancy Rate,” Fertility, Gynecology and Andrology 3, no. 1 (2023): e136798.

[29] I. Thanaboonyawat , S. Pothisan , S. Petyim , and P. Laokirkkiat , “Pregnancy Outcomes After Vaginal Probiotic Supplementation Before Frozen Embryo Transfer: A Randomized Controlled Study,” Scientific Reports 13, no. 1 (2023): 11892. 37482568 10.1038/s41598-023-39078-6 PMC10363539

[30] A. Maleki‐Hajiagha , R. Karimi , S. abbasi , N. Emami , and F. Amidi , “Vaginal Probiotics as Therapeutic Adjuncts for Improving Embryo Transfer Success Rates: A Systematic Review and Meta‐Analysis,” BMC Pregnancy and Childbirth 25, no. 1 (2025): 262, 10.1186/s12884-025-07338-0. 40057712 PMC11890537

[31] A. Huniadi , E. Bimbo‐Szuhai , M. Botea , et al., “Fertility Predictors in Intrauterine Insemination (IUI),” Journal of Personalized Medicine 13, no. 3 (2023): 395. 36983577 10.3390/jpm13030395 PMC10058138

[32] C. Thoda and M. Touraki , “Immunomodulatory Properties of Probiotics and Their Derived Bioactive Compounds,” Applied Sciences 13, no. 8 (2023): 4726.

[33] C. Mazziotta , M. Tognon , F. Martini , E. Torreggiani , and J. C. Rotondo , “Probiotics Mechanism of Action on Immune Cells and Beneficial Effects on Human Health,” Cells 12, no. 1 (2023): cells12010184, 10.3390/cells12010184. PMC9818925 36611977

[34] K. Seif El Dahan , J. Bejjani , A. A. Nasrallah , et al., “Probiotics Properties: A Focus on Pregnancy Outcomes,” European Journal of Obstetrics & Gynecology and Reproductive Biology 272 (2022): 16–23, 10.1016/j.ejogrb.2022.03.008. 35278924

[35] K. O. Kwok , L. R. Fries , I. Silva‐Zolezzi , S. K. Thakkar , A. Iroz , and C. Blanchard , “Effects of Probiotic Intervention on Markers of Inflammation and Health Outcomes in Women of Reproductive Age and Their Children,” Frontiers in Nutrition 9 (2022): 889040. 35734372 10.3389/fnut.2022.889040 PMC9207510

[36] Y. Wang , Y. Wu , Y. Wang , et al., “Antioxidant Properties of Probiotic Bacteria,” Nutrients 9, no. 5 (2017): 521. 28534820 10.3390/nu9050521 PMC5452251

[37] S. M. Dahiphale , D. Dewani , J. M. Dahiphale , et al., “A Comprehensive Review of the Endometrial Receptivity Array in Embryo Transfer: Advancements, Applications, and Clinical Outcomes,” Cureus 16, no. 8 (2024): e67866, 10.7759/cureus.67866. 39328623 PMC11424594

[38] E. Moustakli , S. Stavros , P. Katopodis , et al., “Gut Microbiome Dysbiosis and Its Impact on Reproductive Health: Mechanisms and Clinical Applications,” Metabolites 15, no. 6 (2025): 390. 40559414 10.3390/metabo15060390 PMC12195147

[39] X. Qi , C. Yun , Y. Pang , and J. Qiao , “The Impact of the Gut Microbiota on the Reproductive and Metabolic Endocrine System,” Gut Microbes 13, no. 1 (2021): 1–21, 10.1080/19490976.2021.1894070. PMC7971312 33722164

[40] J. Zeng , Z. He , G. Wang , Y. Ma , and F. Zhang , “Interaction Between Microbiota and Immunity: Molecular Mechanisms, Biological Functions, Diseases, and New Therapeutic Opportunities,” MedComm 6, no. 7 (2025): e70265, 10.1002/mco2.70265. 40547945 PMC12179415

[41] G. Stoyancheva , N. Mihaylova , M. Gerginova , and E. Krumova , “Endometrial Microbiome and Reproductive Receptivity: Diverse Perspectives,” International Journal of Molecular Sciences 26, no. 21 (2025): 10796. 41226831 10.3390/ijms262110796 PMC12609489

